# Multilocus inherited neoplasia allele syndrome (MINAS) in a Turkish cohort: molecular insights and clinical relevance for precision oncology

**DOI:** 10.3389/fphar.2025.1672774

**Published:** 2025-12-19

**Authors:** Taha Bahsi, Serhat Seyhan, Kaan Helvaci, Umut Demirci, Irem Bilgetekin, Selami Bayram, Mehmet Akif Ozturk, Fatih Aydogan, Serkan Keskin, Kezban Pilanci, Nur Sener, Murat Tatlı, Hakan Harputluoglu, Esat Namal, Aysegul Kargi, Mukremin Uysal, Mahmut Ilhan, Cihan Erol, Naziyet Kose, Kerem Okutur, Halit Karaca, Erkan Dogan, Veli Berk, Ahmet Siyar Ekinci, Mustafa Ozdogan

**Affiliations:** 1 Department of Medical Genetics, Üsküdar University, Üsküdar, Türkiye; 2 Memorial Health Group, Ankara, Türkiye; 3 Department of Medical Genetics, MedRoyal Genetic Disease Evaluation Center, Memorial Healthcare Group, Istanbul, Türkiye; 4 Memorial Health Group Antalya, Antalya, Türkiye; 5 Memorial Health Group Istanbul, Istanbul, Türkiye; 6 Department of Medical Genetics, Memorial Bahcelievler Hospital, Istanbul, Türkiye; 7 Memorial Health Group, Istanbul, Türkiye

**Keywords:** MINAS, germline variants, multigene panel, BRCA, Turkish cohort, hereditary cancer

## Abstract

**Background:**

Multilocus Inherited Neoplasia Allele Syndrome (MINAS) describes individuals who harbor pathogenic or likely pathogenic (LP/P) germline variants in two or more distinct cancer predisposition genes. With the broader implementation of next-generation sequencing (NGS) and multigene panel testing, MINAS has been increasingly recognized.

**Methods:**

We retrospectively evaluated 655 Turkish patients referred for hereditary cancer testing using two validated NGS panels. MINAS was defined as the presence of LP/P variants in at least two different genes.

**Results:**

14 patients (2.13%) met the criteria for MINAS. An additional 156 patients (23.8%) had single-gene LP/P variants. MINAS cases accounted for 8.2% of mutation-positive individuals. These patients showed diverse tumor types. Common gene combinations included *BRCA1+MUTYH* and *CHEK2+PALB2*.

**Conclusion:**

This is the first MINAS-focused analysis from a Turkish cohort. Although uncommon, MINAS represents a significant subset of genetically high-risk individuals requiring tailored clinical management.

## Introduction

Hereditary cancer syndromes account for approximately 5%–10% of all malignancies and have long been characterized by monogenic inheritance patterns, wherein a pathogenic variant in a single high-penetrance gene (e.g., *BRCA1*, *BRCA2*, *TP53*) confers a significantly elevated lifetime risk of specific cancers. However, the increasing use of next-generation sequencing (NGS) and the advent of comprehensive multigene panel testing have uncovered a more complex genetic architecture of cancer predisposition than previously understood (Ates et al., 2022; Couch et al., 2017). A growing subset of individuals have been found to harbor pathogenic or likely pathogenic (LP/P) germline variants in more than one cancer susceptibility gene—a phenomenon now referred to as Multilocus Inherited Neoplasia Allele Syndrome (MINAS).

MINAS was first described systematically by Schrader et al., in 2016, who reported individuals carrying LP/P mutations in two or more distinct cancer-associated genes. Variants of uncertain significance (VUS) are not counted toward this definition. Subsequent studies have demonstrated that MINAS is not an exceedingly rare occurrence, with reported prevalence ranging from 0.5% to 3% of individuals undergoing hereditary cancer testing (Whitworth et al., 2016; Agaoglu et al., 2024). These findings have profound implications for clinical practice, including risk assessment, genetic counseling (Hampel et al., 2015), and individualized cancer surveillance and prevention strategies.

Patients with MINAS may exhibit unusual clinical phenotypes, including early age of onset, multiple primary malignancies, or tumors that do not strictly correlate with the phenotype predicted by any one of the individual gene mutations. Additionally, the coexistence of mutations in genes that function within distinct DNA damage response pathways—such as homologous recombination repair (e.g., *BRCA1/2*) and mismatch repair (e.g., *MSH2*, *MSH6*)—may contribute to unique tumor biology and potential therapeutic vulnerabilities (El Tekle et al., 2021). The clinical implications of harboring multiple LP/P variants are still under investigation, and few evidence-based guidelines currently address the optimal management of these patients.

Moreover, MINAS poses significant challenges to genetic counseling. Family histories may appear discordant, with different relatives inheriting distinct variants, complicating risk prediction and cascade testing strategies (Bednar et al., 2024). These complexities are further compounded by limited data on penetrance, expressivity, and the interaction between co-inherited mutations, particularly in underrepresented populations.

Despite increasing recognition of MINAS in the literature, data from non-Western populations remain sparse. Most existing studies originate from North America and Western Europe, leading to a gap in the global understanding of MINAS prevalence and spectrum. Populations with unique genetic backgrounds, such as those in the Middle East and Mediterranean regions, may harbor distinct mutation profiles and gene-gene interactions, underscoring the need for regional studies.

In this context, we present the first comprehensive analysis of MINAS in a Turkish hereditary cancer cohort. Using validated multigene panels, we systematically evaluated 655 individuals for germline LP/P variants and identified those harboring multilocus alterations. Our objectives were to determine the prevalence and spectrum of MINAS in this population, describe the associated tumor phenotypes, and compare our findings with the existing global literature. By expanding the epidemiological and clinical landscape of MINAS, our study contributes to the development of informed guidelines and lays the foundation for future research into the biological and clinical consequences of multilocus inherited cancer risk.

In large cohorts undergoing germline testing, MINAS prevalence ranges between 0.5% and 3% (Tung et al., 2016). These individuals may exhibit complex phenotypes, multiple primary tumors, or early-onset disease. The impact of multiple mutations, particularly across distinct repair pathways like homologous recombination and mismatch repair, remains to be fully elucidated (Drost and Clevers, 2018).

This study presents the first detailed evaluation of MINAS in a Turkish population. We analyzed the frequency, gene combinations, and cancer phenotypes of MINAS individuals within a 655-patient cohort and contextualized these findings in the broader spectrum of hereditary cancer genetics.

## Materials and methods

### Study population and ethical approval

This retrospective study included 655 consecutive patients who were referred for germline hereditary cancer testing between February 2024 and May 2025 at tertiary medical genetics clinics within the Memorial Healthcare Group, Turkey. Eligible individuals had a personal and/or family history suggestive of hereditary cancer syndromes, including early-onset malignancies, multiple primary tumors, and/or a significant family history of cancer in first- or second-degree relatives. Patients with insufficient clinical data or inadequate DNA quality were excluded.

All procedures were conducted in accordance with the Declaration of Helsinki. Ethical approval was obtained from the Antalya Memorial Hospital Ethics Committee (Decision No: 06, Date: 18.06.2025). Since all data were de-identified and used retrospectively, individual informed consent was not required according to institutional policy.

### DNA isolation and library preparation

Peripheral blood samples were collected in EDTA tubes, and genomic DNA was extracted using the QIAamp DNA Blood Mini Kit (Qiagen) and the Promega Maxwell RSC Blood DNA Kit according to the manufacturers’ protocols. DNA quantity and purity were assessed using NanoDrop spectrophotometry and Qubit fluorometric quantification.

### Next-generation sequencing and bioinformatics

Sequencing data were processed using vendor-specific pipelines. Raw reads were subjected to base calling, demultiplexing, and adapter trimming. Sequences were aligned to the human reference genome (GRCh37/hg19) using BWA-MEM. Sequencing was performed on the Illumina NextSeq 2000 platform following the manufacturer’s standard workflow. Sophia Genetics Hereditary Cancer Solutions Panel was used with cancer predisposition genes i*ATM, APC, AXIN2, BAP1, BARD1, BLM, BMPR1A, BRCA1, BRCA2, BRIP1, CDH1, CDK4, CDKN2A, CHEK2, EPCAM, DDB2, ERCC2, ERCC3, ERCC4, ERCC5, FANCA, FANCC, FH, FLCN, GALNT12, HDAC2, HOXB13, MEN1, MET, MITF, MLH1, MSH2, MSH3, MSH6, MUTYH, NBN, NF1, NF2, NTHL1, PALB2, PMS2, PMS2CL, POLD1, POLE, POLH, PTCH1, PTEN, RAD51C, RAD51D, RB1, RET, SMAD4, STK11, TP53, TSC1, TSC2, VHL, WT1, XPA, XPC*. Libraries passing initial quality control were loaded onto the NextSeq 2000 flow cell using the integrated loading system. Cluster generation and sequencing were carried out on-board with a paired-end read configuration (2 × 150 bp). Primary data processing, including base calling and quality scoring, was performed in real time using Sophia DDM™ cloud platform. Raw BCL files were subsequently demultiplexed and converted to FASTQ format for downstream analysis. Also other targeted sequencing was performed using the Twist Bioscience Hereditary Cancer Panel, which includes a comprehensive set of cancer predisposition genes *ABL1, ABRAXAS1, AKT1, ALK, ALX1, APC, AR, ATM, AXIN2, BAP1, BARD1, BLM, BMPR1A, BRAF, BRCA1, BRCA2, BRIP1, CALR, CASP8, CBL, CDH1, CDK4, CDKN2A, CEBPA, CHEK2, CSF1R, CSF3R, CTNNB1, DICER1, DIS3L2, DNM2, DNMT3A, EGFR, ELA2, ELANE, EPCAM, ERBB2, ERBB4, ESR1, EZH2, FANCA, FANCB, FANCC, FANCD2, FANCE, FANCF, FANCG, FANCI, FANCL, FANCM, FBXW7, FGFR1, FGFR2, FGFR3, FH, FLCN, FLT3, GALNT12, GFI1, GNA11, GNAQ, GNAS, HAX1, HMMR, HNF1A, HNF1B, HOXB13, HRAS, IDH1, IDH2, JAK2, JAK3, KDR, KIT, KRAS, MC1R, MEN1, MET, MITF, MLH1, MLH3, MPL, MRE11, MRE11A, MSH2, MSH3, MSH6, MUTYH, MUTYH, NBN, NF1, NOTCH1, NPM1, NQO2, NRAS, NTHL1, PALB2, PDGFRA, PHB, PIK3CA, PMS1, PMS2, POLD1, POLE, POT1, PPM1D, PRSS1, PTCH1, PTEN, PTPN11, RAD50, RAD51, RAD51C, RAD51D, RAD54L, RB1, RB1CC1, RECQL, RET, RNF43, RRAS2, RUNX1, SDHA, SDHAF2, SDHB, SDHC, SDHD, SETBP1, SF3B1, SLC22A1L, SLX4, SMAD4, SMARCA4, SMARCB1, SMO, SRC, SRSF2, STK11, TET2, TGFBR2, TP53, TSC1, TSC2, U2AF1, USB1, VHL, WAS, WT1, XRCC2, XRCC3*. Library preparation was carried out according to the manufacturer’s protocol, followed by circularization and generation of DNA nanoballs (DNBs). Sequencing was conducted on the MGI DNBSEQ-G400 platform using paired-end reads (2 × 150 bp) with combinatorial Probe-Anchor Synthesis (cPAS) chemistry. Raw data were processed through MGI’s standard pipeline for base calling, demultiplexing, and adapter trimming. Reads were aligned to the human reference genome (GRCh37/hg19) using BWA-MEM. Variant calling and downstream analysis were performed using the GenomizeSeq clinical bioinformatics pipeline.

Single nucleotide variants (SNVs) and small insertions/deletions (indels) were annotated and classified according to the 2015 ACMG-AMP guidelines. Copy number variants (CNVs) were evaluated using depth-of-coverage–based algorithms embedded within the GenomizeSeq platform. All pathogenic (P) and likely pathogenic (LP) variants, along with variants of uncertain significance (VUS), were reviewed and manually curated in a clinical context. The final report included detailed variant annotation, zygosity, ACMG classification, associated phenotypes, and clinical actionability, if applicable.

Quality control thresholds included minimum coverage of ≥100× and variant allele frequency (VAF) ≥0.2 for heterozygous variants.

### Variant annotation and classification

Variants were annotated using the Genomize Seq Platform and classified following the ACMG/AMP 2015 guidelines. Classifications were cross-referenced with ClinVar, gnomAD, HGMD Professional, and LOVD databases. Only pathogenic or likely pathogenic (LP/P) variants were included in the final analysis. Variants of uncertain significance (VUS) were excluded. Zygosity was determined by examining VAF and sequencing reads.

### Definition of MINAS and clinical data collection

Multilocus Inherited Neoplasia Allele Syndrome (MINAS) was defined as the presence of LP/P germline variants in ≥2 distinct cancer predisposition genes in the same individual. Compound heterozygous or homozygous variants in the same gene (e.g., biallelic *MUTYH*) were not classified as MINAS unless accompanied by a second LP/P variant in a different gene.

Clinical data including tumor type, age at diagnosis, and family history were retrospectively extracted from electronic medical records. Where available, pedigree charts and pathology reports were reviewed by board-certified clinical geneticists. Tumor phenotype–genotype concordance was assessed using NCCN and ESMO guidelines.

### Statistical analysis

Descriptive statistics were used to report the frequency of MINAS cases and associated gene combinations. Categorical variables were expressed as counts and percentages, and continuous variables as medians with interquartile ranges (IQR). Comparisons between groups (e.g., MINAS vs. non-MINAS) were conducted using Fisher’s exact test for categorical variables and the Mann–Whitney U test for continuous variables. A p-value <0.05 was considered statistically significant. All analyses were conducted using R version 4.2.2.

## Results

**FIGURE 1:**
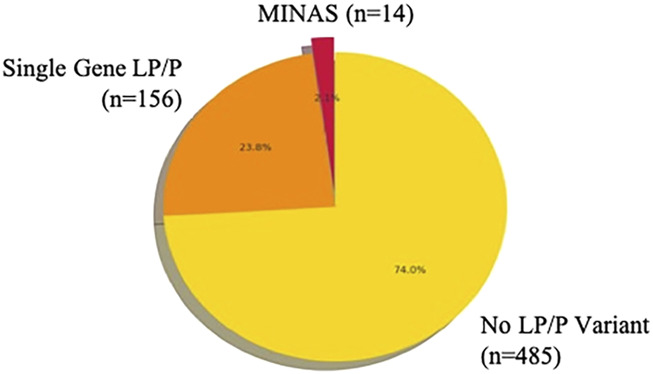
Distribution of LP/P variants in the MINAS and non-MINAS subgroups.

A total of 655 patients underwent germline genetic testing using either the Twist and Sophia hereditary cancer panels. Of these, 170 individuals (26%) were found to carry at least one pathogenic or likely pathogenic (LP/P) variant. Among mutation-positive patients, 156 (23.8% of total) had LP/P variants in a single gene, while 14 patients (2.13% of the cohort) met the criteria for MINAS, defined as the presence of LP/P variants in two or more distinct cancer predisposition genes. Consequently, MINAS accounted for 8.2% of the mutation-positive group (14/170) (Figure 1).

### Clinically significant mutation frequency in non-MINAS cohort

**FIGURE 2:**
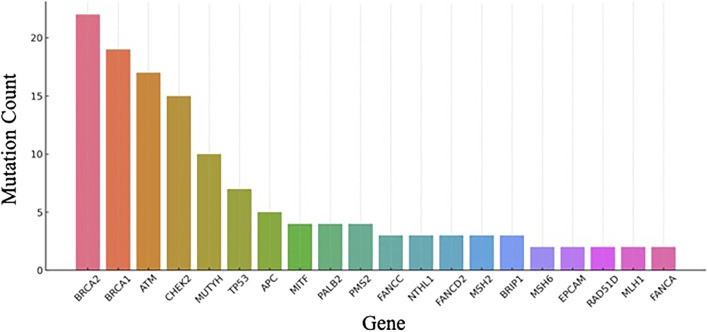
Gene-wise distribution of pathogenic and likely pathogenic variants in non-MINAS patients (bar chart).

This bar graph (Figure 2) illustrates the frequency of clinically significant variants (Pathogenic, Likely Pathogenic, and Risk Factor variants) across genes in the non-MINAS cohort. The data was extracted from hereditary cancer panel testing results and reflects mutation burden distribution among relevant cancer predisposition genes. Only variants explicitly classified as pathogenic, likely pathogenic, or noted as a risk factor were included.

**FIGURE 3:**
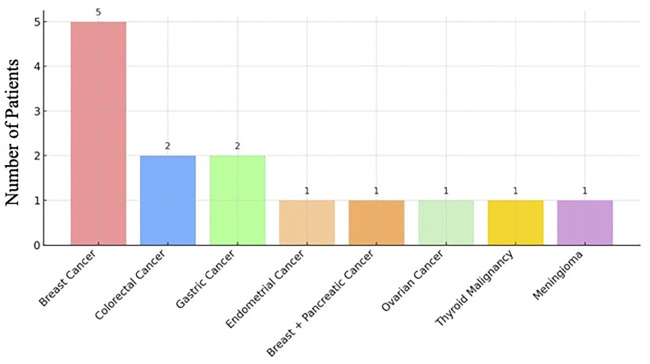
Tumor type distribution among patients with MINAS.

When stratified by tumor type, the most common cancer phenotype among MINAS patients was breast cancer, observed in 5 out of 14 cases (36%). This was followed by colorectal cancer in 2 patients, gastric cancer in 2 patients (14%), ovarian cancer in 1 patient (7%), endometrial cancer in 1 patient (7%), thyroid malignant neoplasm in 1 patient (7%), meningioma in 1 patient (7%) and co-occurrence of breast and pancreatic cancer in 1 patient (Figure 3). Notably, some patients exhibited phenotypic features consistent with both mutated genes, whereas others presented with tumors typically associated with only one of the implicated genes.

Analysis of the genetic findings in MINAS cases revealed that the most frequently involved genes were *BRCA1, BRCA2, MUTYH, CHEK2, PALB2,* and *MSH6.* Gene combinations observed more than once included *BRCA1+MUTYH* and *CHEK2+PALB2*. Importantly, all patients with co-occurring mutations had variants interpreted as LP or P according to ACMG guidelines, supported by ClinVar classification.

In terms of variant type, the majority were frameshift mutations followed by missense, nonsense and splice-site variants. The zygosity status was predominantly heterozygous for all detected variants, consistent with dominant inheritance patterns typical of high-risk cancer susceptibility genes. No biallelic or homozygous configurations were included in the MINAS categorization.

Among all LP/P variant carriers (n = 170), the presence of multiple mutations (MINAS) was significantly less frequent than single-gene alterations, but their clinical implications may be disproportionately impactful due to potential synergistic or additive effects on tumorigenesis. Detailed clinical and genetic characteristics of each MINAS patient are provided in Table 1.

**TABLE 1:** Clinical and genetic characteristics of patients with MINAS.

Case	Sex	Age	Clinical diagnosis	Gene/Transcript	Variant/Protein change	Zygosity	Classification	Inheritance	Gene–Disease validity
1	F	61	Ovarian cancer	*BRCA1 (NM_007294.3)*	c.5266dup > G p.(Gln1756Profs*74)	Het	Pathogenic	AD	Definitive (HBOC)
				*ERCC3 (NM_000122.1)*	c.1421dup p.(Asp474Glufs*2)	Het	Pathogenic	AR	Moderate (Xeroderma pigmentosum; DNA repair; cancer risk evolving)
2	F	49	Breast cancer	*BRCA1 (NM_007294.3)*	c.1504_1508del p.(Leu502Ala fs*)	Het	Pathogenic	AD	Definitive
				*MUTYH (NM_001048171.1)*	c.760C>T p.(Gln254*)	Het	Pathogenic	AR	Definitive (biallelic CRC); Limited (monoallelic)
3	F	39	Breast + pancreatic ca	*BRCA1 (NM_007294.3)*	c.4986 + 6T>C p.(?)	Het	Pathogenic	AD	Definitive
				*PALB2 (NM_024675.3)*	c.173del p.(LeuCysfs*10)	Het	Pathogenic	AD	Definitive (breast/pancreatic cancer)
4	M	71	Thyroid malignant neoplasm	*CHEK2 (NM_001005735.1)*	c.721 + 3A>T p.(?)	Het	Likely pathogenic	AD	Strong/Moderate (breast, thyroid, CRC risk)
				*MSH3 (NM_002439.4)*	c.2254-1_2257delinsTAC p.(?)	Het	Likely pathogenic	AR	Limited/Moderate (biallelic CRC)
5	F	52	Breast cancer	*NTHL1 (NM_002528)*	c.244C>T p.(Gln82Ter)	Het	Pathogenic	AR	Definitive (biallelic CRC); Limited (monoallelic)
				*JAK3 (NM_000215)*	c.1744C>T p.(Arg582Trp)	Het	Pathogenic	AD	Limited (hematological malignancies, evolving)
6	F	39	Endometrial ca	*MSH2 (NM_000251)*	c.1276 + 1G>T p.(?)	Het	Pathogenic	AD	Definitive (Lynch syndrome)
				*RAD51C (NM_058216)*	c.706–2A>G p.(?)	Het	Pathogenic	AD	Definitive (hereditary breast/ovarian cancer)
7	F	49	Breast ca	*FANCM (NM_020937.4)*	c.5101C>T p.(Gln1701Ter)	Het	Pathogenic	AR	Moderate (biallelic FA; monoallelic breast ca risk evolving)
				*RAD54L (NM_003579.4)*	c.475G>T p.(Glu159Ter)	Het	Likely pathogenic	AR	Limited (DNA repair; cancer risk evolving)
8	M	28	Colorectal ca	*MLH1 (NM_000249.3)*	c.2073dup p.(Ser692IlefsTer2)	Het	Likely pathogenic	AD	Definitive (Lynch syndrome)
				*CHEK2 (NM_001005735.2)*	c.190G>A p.(Glu64Lys)	Het	Likely pathogenic	AD	Strong/Moderate
9	F	48	Breast ca	*NTHL1 (NM_002528.7)*	c.244C>T p.(Gln82Ter)	Het	Pathogenic	AR	Definitive (biallelic); Limited (monoallelic)
				*PPM1D (NM_003620.4)*	c.185_186del p.(Glu62GlyfsTer27)	Het	Likely pathogenic	AD	Moderate (therapy-related clonal hematopoiesis, evolving)
10	F	39	Breast ca	*NF1 (NM_0010424923)*	c.5268 + 5G>T p.(?)	Het	Likely pathogenic	AD	Definitive (Neurofibromatosis type 1, breast ca risk)
				*MUTYH (NM_012222.2)*	c.1178G>A p.(Gly393Asp)	Het	Likely pathogenic	AR	Definitive (biallelic CRC); Limited (monoallelic)
11	M	57	Gastric ca	*MSH2 (NM_000251.3)*	c.1204C>T p.(Gln402Ter)	Het	Pathogenic	AD	Definitive (Lynch)
				*FANCA (NM_000135.4)*	c.4124_4125del p.(Thr1375SerfsTer49)	Het	Pathogenic	AR	Definitive (biallelic FA); Limited (monoallelic cancer risk evolving)
12	M	28	Colorectal ca	*ATM (NM_000051.3)*	c.5762 + 1G>T p.(?)	Het	Pathogenic	AD	Definitive/Strong (breast, pancreatic, CRC)
				*FANCC (NM_000136.3)*	c.521 + 1G>A p.(?)	Het	Pathogenic	AR	Definitive (biallelic FA); Limited (monoallelic)
13	M	67	Gastric ca	*MSH2 (NM_000251.3)*	c.1667T>C p.(Leu556Ser)	Het	Pathogenic	AD	Definitive
				*PMS2 (NM_000535.7)*	c.1012C>T p.(Pro338Ser)	Het	Likely pathogenic	AD	Definitive
14	F	34	Meningioma	*ATM (NM_000051.3)*	c.4909 + 1G>A p.(?)	Het	Pathogenic	AD	Strong/Definitive
				*MITF (NM_001354604.2)*	c.1273G>A p.(Glu425Lys)	Het	Pathogenic	AD	Moderate (melanoma, renal cell carcinoma; evolving in brain tumors)

These findings reinforce the diagnostic and clinical value of comprehensive multigene panel testing, especially in genetically heterogeneous populations, and suggest that MINAS may be underrecognized in standard single-gene approaches. Tumor types in MINAS patients included breast cancer (n = 5), colorectal cancer (n = 2), gastric cancer (n = 2), endometrial cancer (n = 1), co-occurrence of breast and pancreatic cancer (n = 1), ovarian cancer (n = 1), thyroid malignant neoplasm (n = 1), meningioma (n = 1). Variants most frequently involved *BRCA1, BRCA2, MUTYH, CHEK2, PALB2*, and *MSH6.* Among 14 MINAS patients, common gene combinations included *BRCA1+MUTYH* and *CHEK2+PALB2*.

As this study involves sensitive patient data, the datasets are not deposited in public repositories. The datasets generated and analyzed during the current study are available from the corresponding author upon reasonable request.

## Discussion

This study presents the first comprehensive evaluation of MINAS in a Turkish hereditary cancer cohort and contributes to the growing recognition that multilocus germline mutations are not rare among genetically predisposed individuals (Schrader et al., 2016; Whitworth et al., 2016; Agaoglu et al., 2024). We observed a MINAS prevalence of 2.1% in the overall cohort and 8.2% among mutation-positive patients, in line with previously reported rates ranging from 0.5% to 3%.

The coexistence of pathogenic variants in distinct cancer susceptibility genes may affect disease onset, spectrum, and progression. In our study, multiple MINAS patients developed cancer before the age of 50, and some exhibited tumor types associated with both mutated genes—such as the combination of *BRCA1* and *MUTYH*, suggesting additive or synergistic effects (El Tekle et al., 2021). With respect to recessive genes such as MUTYH, we agree that the role of monoallelic carriers is still debated. However, consistent with prior literature, we have included these variants when they co-occurred with a second pathogenic variant in a definitive cancer susceptibility gene, as such cases may still contribute to understanding the broader phenotypic spectrum of MINAS.

Genetic counseling for MINAS remains challenging. Pedigree analysis often fails to capture the complexity of multilocus inheritance, and risk stratification may be incomplete without multigene panel testing (Bednar et al., 2024; Hampel et al., 2015). Current clinical guidelines primarily focus on monogenic inheritance models (e.g., *BRCA1*, *TP53*), and do not yet fully address how to manage individuals with multiple LP/P variants. Developing risk-adapted surveillance and preventive care strategies for such cases should be a priority for expert panels such as NCCN and ESMO.

Therapeutically, MINAS cases may open novel windows of opportunity. For example, individuals with homologous recombination deficiency (HRD) due to *BRCA* mutations may benefit from PARP inhibitors (Mirza et al., 2016), while coexisting mismatch repair (MMR) deficiency (e.g., *MSH2*, *PMS2*) may make them eligible for immunotherapy (Le et al., 2017). The biological consequences of these combinatorial genotypes remain underexplored, but preclinical modeling—especially through organoid-based systems—may help illuminate these interactions (Drost and Clevers, 2018).

The clinical case involving an *ATM*-*MITF* dual variant carrier identified during segregation analysis further emphasizes the diagnostic power of multigene testing. In this context, large panel-based approaches not only enhance mutation detection rates but also generate clinically meaningful insights (Mandelker et al., 2019), particularly in genetically heterogeneous populations like Turkey (Ates et al., 2022).

A key limitation of this study is its retrospective design and reliance on available clinical data for phenotype-genotype correlation. Moreover, segregation analysis and functional assays were not conducted, which limits inference on variant expressivity and penetrance (Kingdom and Wright, 2022).

In cases of MINAS (Multilocus Inherited Neoplasia Allele Syndrome), clinical follow-up, preventive strategies, and surveillance are often more challenging due to the presence of pathogenic variants in two distinct cancer predisposition genes. This complexity arises from the fact that the penetrance, cancer spectrum, and tissue/organ specificity associated with each gene can differ significantly. As a result, it is often unclear whether a malignancy observed in a patient originates primarily from one gene, or represents a synergistic effect of both variants—with uncertainty regarding which gene exerts a dominant influence. Therefore, detailed family segregation analysis and phenotypic assessment of other family members are critically important—not only for the follow-up of the index case but also for the clinical management of other MINAS carriers or monoallelic mutation carriers within the family.

In summary, MINAS represents a distinct and clinically relevant subgroup of hereditary cancer syndromes. As precision oncology advances, dedicated efforts are needed to understand the biological and clinical implications of multilocus germline variation and to design personalized management strategies accordingly.

## Conclusion

Our study expands the clinical and genomic landscape of MINAS by characterizing its prevalence, gene combinations, and tumor phenotypes in a Turkish hereditary cancer cohort. The detection of MINAS in 2.1% of tested individuals highlights the utility of multigene panel testing in uncovering complex germline architectures. Recognizing and systematically evaluating MINAS is essential for advancing precision prevention (McGuigan et al., 2022), risk counseling, and tailored surveillance strategies in hereditary cancer care.

## Data Availability

The original contributions presented in the study are publicly available. This data can be found here: NCBI repository, accession number PRJNA1390191.
